# Clinical study on the treatment of acute epidural hematoma by embolization of middle meningeal artery through radial artery access combined with minimally invasive burr hole drainage

**DOI:** 10.3389/fneur.2025.1502408

**Published:** 2025-02-07

**Authors:** Cui Zhang, Qingbo Wang, Chenglong Li, Zixuan Jing, Xinyu Zhao, Yang Chen, Zefu Li

**Affiliations:** Department of Neurosurgery, Binzhou Medical University Hospital, Binzhou, Shandong, China

**Keywords:** acute epidural hematoma, middle meningeal artery embolization, endovascular interventional embolization therapy, distal radial artery access, burr hole drainage, intracranial hematoma removal

## Abstract

**Objectives:**

This study aims to evaluate the clinical effectiveness of using coil embolization via radial artery access, combined with drilling and drainage, as a minimally invasive treatment for acute epidural hematoma compared to traditional craniotomy.

**Materials and methods:**

A retrospective analysis was conducted on 134 patients with acute epidural hematoma treated at the Department of Neurosurgery, Binzhou Medical University Hospital, between January 2020 and April 2023. Among these patients, 37 underwent embolization of the middle meningeal artery through radial artery access combined with burr hole drainage, while 97 patients underwent craniotomy for hematoma removal. The 37 patients treated with embolization were designated as the experimental group, while the remaining 97 patients formed the control group. General patient characteristics, operation duration, intraoperative blood loss, postoperative complications, and Glasgow Coma Scale (GCS) scores upon admission and discharge were recorded and compared between the two groups.

**Results:**

In comparison to the control group, the experimental group exhibited higher rates of postoperative hematoma residuals and longer average postoperative drainage times. Nevertheless, the experimental group demonstrated several advantages including shorter operation durations, reduced intraoperative bleeding, lower rates of postoperative rebleeding and complications, as well as decreased requirements for postoperative blood transfusions and transfusion volumes.

**Conclusion:**

The surgical approach involving coil embolization via radial artery access combined with minimally invasive burr hole drainage yields favorable clinical outcomes. This technique presents as a viable treatment option for acute epidural hematoma resulting from middle meningeal artery hemorrhage.

## 1 Introduction

Acute epidural hematoma (AEDH) is a common and perilous secondary injury in craniocerebral trauma. Rapidly expanding hematomas can compress the brain within hours, posing a life-threatening risk to patients (1, 2). Treatment for AEDH now includes both conservative and surgical approaches. Patients with small hematomas and stable conditions may undergo conservative management. However, regular monitoring with CT scans and continuous observation of the patient’s status are crucial. Surgery becomes necessary if conservative treatment fails. Urgent craniotomy is performed when the volume of temporal hematomas exceeds 20 mL or the total hematoma volume exceeds 30 ml (3). While craniotomy yields better outcomes, it carries higher invasiveness and postoperative complications (4, 5). Patients may experience negative psychological effects such as anxiety and depression post-surgery, affecting their familial and social life. Consequently, many neurosurgeons are advocating for minimally invasive approaches to craniocerebral injuries. Among these, drill drainage is regaining popularity due to its minimal trauma. In cases of intracranial hypertension, burr hole drainage can be performed urgently to buy time for further treatment. However, acute epidural hematomas are primarily caused by the rupture of the middle meningeal artery (MMA), leading to substantial bleeding and unstable hematoma. Drill drainage alone cannot effectively control bleeding, limiting its utility in AEDH. Endovascular intervention has seen rapid advancements across various medical fields, including neurological disorders such as arterial stenosis, aneurysms, and vascular abnormalities (6–8). Access options for intervention continually evolve to improve patient comfort and reduce the burden of care provision. Transradial artery access (TRA) has emerged as a preferred approach for diagnosing and treating coronary artery disease due to its enhanced comfort and safety. TRA is gradually being adopted in cerebrovascular procedures and the management of cerebrovascular disorders (9, 10). Some studies suggest that cerebral angiography and embolization of bleeding vessels can accurately identify bleeding sources and achieve effective hemostasis (11, 12). Therefore, we propose that combining drill drainage with MMA embolization via TRA is an efficient and safe therapy for acute epidural hematoma.

## 2 Materials and methods

### 2.1 Patient information

Between January 2020 and April 2023, a total of 134 patients diagnosed with AEDH were enrolled in this study at the neurosurgery division of Binzhou Medical University Hospital. The surgical method was chosen by the patient themselves. Among them, 37 patients were assigned to the experimental group, receiving spring coil embolization of the MMA via TRA, combined with drilling and drainage. The control group comprised 97 patients who underwent traditional open craniotomy for hematoma removal. Detailed patient characteristics, procedural duration, intraoperative blood loss, postoperative complications, GCS scores at admission and discharge, and other relevant parameters were meticulously documented and compared between the two groups.

Patient inclusion requirements:

1. Patients presenting with cranial CT evidence of epidural hematoma in the frontal, temporal, frontoparietal, and temporoparietal regions.
2. Patients who underwent MMA embolization via TRA, in combination with drilling and drainage, and met the surgical criteria (AEDH patients with hematoma volume > 30 mL or temporal hematoma volume > 20 mL).
3. Admission within 24 h of trauma.
4. Initial GCS score ranging from 11 to 15, with worsening clinical symptoms such as headache, nausea, and vomiting over time.

Patient Exclusion requirements:

1. Patients with normal visualization of MMA on cerebral angiography, occipital epidural hematoma, epidural hematoma in the posterior cranial fossa, suspected cranial venous sinus hemorrhage, or any of these conditions.
2. Patients exhibiting cerebral herniation.
3. Patients with known contrast allergy.
4. Patients with concomitant severe heart, liver, or kidney disease.

### 2.2 Surgical procedure

#### 2.2.1 AEDH removal with open craniotomy

Upon successful induction of general anesthesia, the patient is placed in the supine position, and surgical site disinfection is performed. The choice of surgical incision is determined by the location and shape of the hematoma. Sequential incisions are made through all layers of the skin, ensuring thorough hemostasis. Upon exposure of the skull, an electric drill is used to create perforations, followed by a milling cutter to create a bone flap tailored to the size of the hematoma. The dura mater is carefully elevated, and the epidural hematoma is excised. Concurrently, an electric knife is employed to achieve hemostasis of the branches of the middle meningeal artery (MMA). Upon successful removal of the hematoma, hemostasis is reassured, the surgical site is irrigated with warm saline, and inspected for any signs of ongoing bleeding. The bone flap is repositioned and secured with two connecting pieces and four titanium nails, followed by progressive closure of the layers. A silicone drainage tube with side apertures is placed in the epidural space. Post-procedurally, regular intervals of CT scanning are conducted, and the drainage tube is removed when deemed appropriate.

#### 2.2.2 Embolization of the MMA by TRA in conjunction with minimally invasive drilling and drainage for AEDH

**Figure 1:**
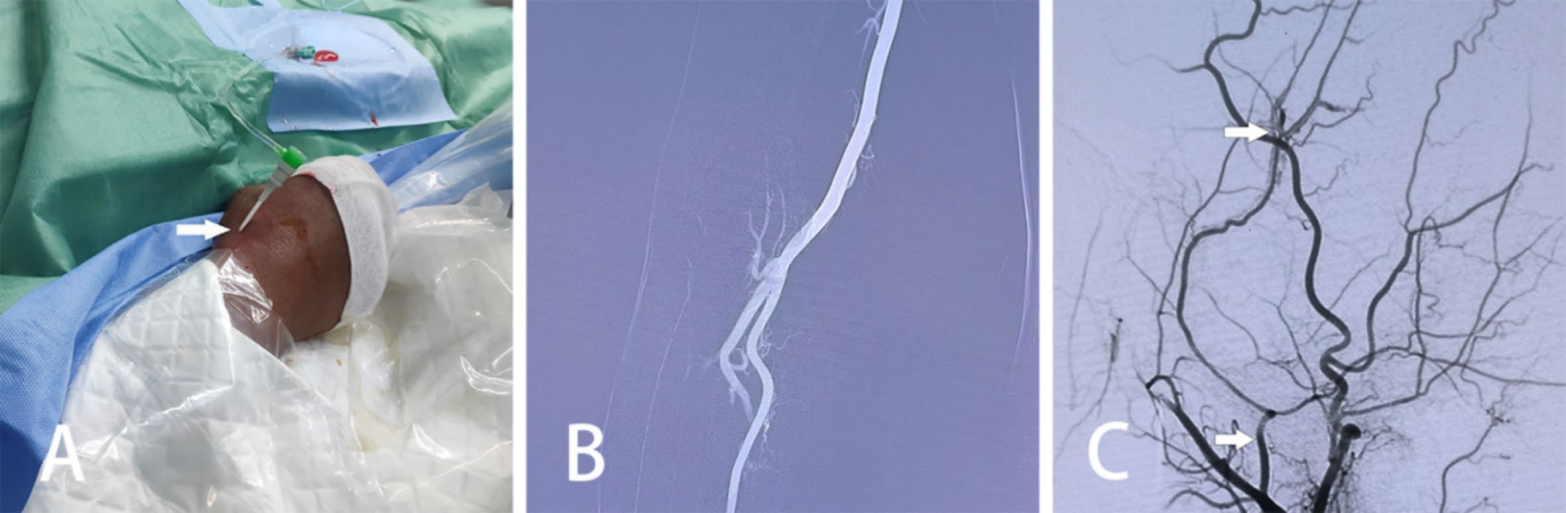
Intraoperative pictures and images of MMA embolization via TRA in patients with AEDH. **(A)** Utilization of the Seldinger technique to access the right radial artery through a puncture in the anatomical snuffbox. **(B)** Radial arteriography of the right arm conducted. **(C)** DSA displaying contrast spillage distal to the MMA (arrows indicate the MMA, as well as the site of contrast spillage).

**Figure 2:**
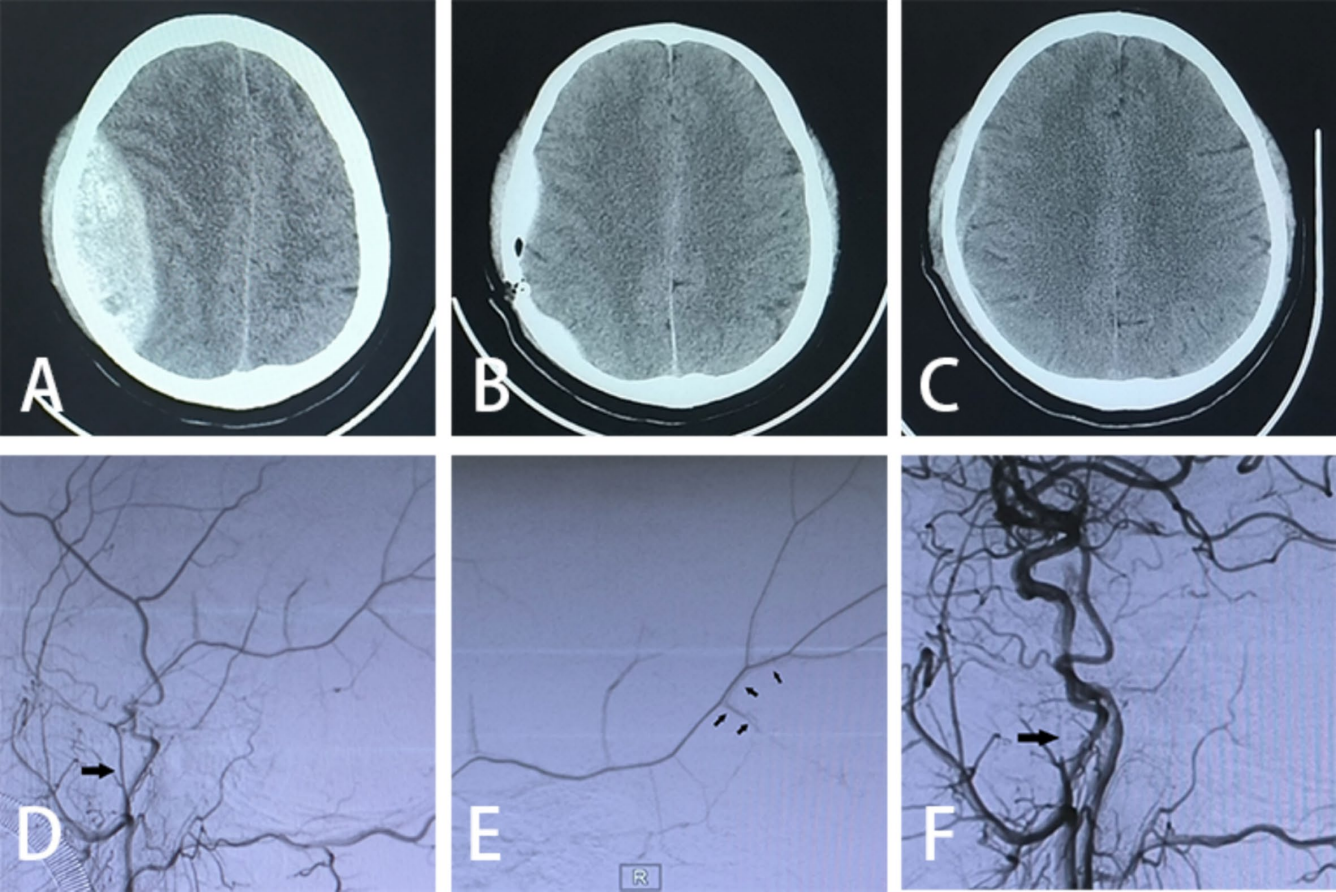
Imaging data. **(A)** Preoperative cranial CT scan; **(B)** postoperative cranial CT scan on day 1; **(C)** postoperative cranial CT scan on day 21; **(D)** preoperative DSA exhibiting MMA abnormality (large arrows); **(E)** intraoperative DSA demonstrating contrast spillage (small arrows); **(F)** postoperative DSA indicating undeveloped MMA (large arrows pointing to the spring-coil embolization).

The patient is positioned supine, and a combination of intravenous and inhalation anesthesia is administered through endotracheal intubation to ensure effective anesthesia during surgery. The procedure consists of two main components: (1) MMA Embolization via TRA: Before commencing the procedure, a thorough preoperative assessment of the radial artery is conducted to identify any potential issues. A 6F radial artery sheath is inserted, followed by the utilization of a 5F catheter system to map vascular access. Subsequently, cerebral angiography and embolization are performed. The patient’s right hand and forearm are sterilized routinely, and the distal radial artery is punctured using the Seldinger method through the anatomical snuffbox of the right hand (Figure 1A). Once the 6F radial artery catheter sheath is successfully positioned, a Y-valve and a three-way tube are connected, with a lateral tube attached to pressured drip. Imaging is conducted using a 5F sim2 contrast catheter under guidewire guidance. Radial artery angiography is initially performed in the right arm to establish a radial artery roadmap (Figure 1B). The guidewire is advanced along with the catheter until it reaches under the right common carotid artery, where cranial digital subtraction angiography (DSA) is performed. DSA reveals contrast spillage at the MMA (Figure 1C). Subsequently, a microguidewire with a microcatheter is navigated into the MMA, and embolization of the main trunk of the MMA is carried out using Tong Qiao Feng series spring coils (2 mm × 8 mm), EV3 spring coils (2 mm × 8 mm), or Microplex (2 mm × 8 mm). Post-embolization, DSA is reviewed, confirming the invisibility of MMA vessels above the foramen spinosum and their branch vessels. The puncture site of the right radial artery is closed, and the microcatheter and arterial sheath are removed following successful embolization. (2) Minimally Invasive Drilling and Drainage: Preoperative Xper CT is performed to determine the location for hematoma puncture. A 3 cm skin incision is made at the puncture site, followed by drilling two burr and creating a 2 cm by 2 cm bone window. A thicker, porous drain is inserted to evacuate the epidural hematoma in all directions once identified as a clot through the bone window. A new epidural drain is then inserted, and a negative pressure ball is connected. Each layer is sutured sequentially. Urokinase (20,000 U dissolved in 2 mL saline, administered twice daily) is injected at the beginning of postoperative day 1. Periodic postoperative CT examinations are conducted, and the drainage tube is withdrawn as needed. The relevant imaging data for patients in the experimental group are depicted in Figure 2.

### 2.3 Clinical outcomes and follow-up

The safety and efficacy of middle meningeal artery embolization in conjunction with drilling and drainage were evaluated based on intraoperative and postoperative parameters. Intraoperative measures included operative time and intraoperative blood loss. Postoperative assessments encompassed the following: duration of hospitalization, GOS score at 30 days postoperatively, residual hematoma volume assessed by repeat CT scan performed 6 h after surgery (calculated as: Hematoma Volume ml = length × width × layer spacing × number of layers × 0.5 cubic centimeters), duration of postoperative drainage, postoperative rebleeding rate, postoperative complication rate, GCS score at discharge, and volumes of intraoperative and postoperative blood transfusions. The imaging results were compared and evaluated using preoperative cranial CT, 6-h postoperative cranial CT, and 1 month postoperative cranial CT.

### 2.4 Statistical analysis

Data analysis was performed using SPSS version 28.0. Continuous variables with non-normal distributions were characterized using median and quartiles, and between-group comparisons were made using non-parametric tests. Categorical variables were described using frequencies and percentages, with between-group comparisons conducted using the chi-square test. In instances where categorical variables did not meet the requirements for the chi-square test, Fisher’s exact probability test was utilized. Statistical significance was set at *p* < 0.05.

## 3 Results

### 3.1 General clinical data

Table 1 presents an overview of the pre-treatment demographic characteristics of the patients in both groups. A total of 134 patients were included in the data collection, with 37 cases in the experimental group and 97 cases in the control group. Among the experimental group, 32 cases exhibited clinical symptoms of persistent unremitting headache or progressive aggravation, 28 cases of dizziness, 14 cases of vomiting, and 11 cases of impaired consciousness. Similarly, in the control group, 83 cases showed symptoms of persistent unremitting headache or progressive aggravation, 86 cases of dizziness, 35 cases of vomiting, and 24 cases of impaired consciousness. Statistical analysis revealed no significant age difference between the experimental group (40.00, 65.00) and the control group (32.00, 67.00) (*p* > 0.05). Likewise, no significant differences were observed between the experimental and control groups regarding gender, GCS score at admission, underlying illness, duration of disease onset, or symptoms upon admission.

**Table 1:** General clinical data.

Targets	Experimental group (*n* = 37)	Control group (*n* = 97)	*p*	*z/χ*^2^/Fisher
Age, Median (IQR)	53.0(39.5,65.5)	52.0(32.0,67.5)	0.464	−0.732
Gender, *n*(%)			0.128	2.312
Male	27(72.97)	57(58.76)		
Female	10(27.03)	40(41.24)		
Period of disease start, Median (IQR)	2.0(1.5,3.0)	2.0(1.3,3.0)	0.750	−0.319
GCS score at admission, Median (IQR)	13.0(12.0,15.0)	13.0(12.0,14.0)	0.481	−0.705
Underlying illness, *n*(%)				
Hypertension	8(21.62)	21(21.65)	0.997	0.000
Diabetes	7(18.92)	9(9.28)	0.124	2.367
Coronary artery atherosclerotic heart disease	3(8.11)	3(3.09)	0.209	1.575
Symptom, *n*(%)				
Headache	32(86.49)	83(85.57)	0.891	0.019
Dizzy	28(75.68)	86(88.66)	0.059	3.566
Vomit	14(37.84)	35(36.08)	0.850	0.036
disorder of consciousness	11(29.73)	24(24.74)	0.557	0.345

There was no statistically significant difference between the experimental and control groups concerning age, gender, GCS score at admission, underlying illness, period of disease onset, or symptoms upon admission.

### 3.2 Preoperative imaging data

Table 2 presents descriptive statistics of the imaging data for both groups and a comparative analysis. It is evident that the frontal and temporal regions are primarily affected by acute epidural hematomas. In the experimental group, 9 patients (24.32%) had hematomas in the frontal region and 12 patients (32.43%) in the temporal region, with hematoma volumes of 40.0 (36.5, 46.0) ml, midline shift degrees of 0.44 (0.3, 0.6) cm, and hematoma thicknesses of 6.00 (4.8, 7.8) cm. Additionally, among the 37 patients, craniofractures were observed in 37 cases, subdural hematomas in 12 cases, subarachnoid hemorrhages in 10 cases, and cerebral contusions in 25 cases. In the control group, 32 patients (32.99%) had hematomas in the frontal region and 34 patients (35.05%) in the temporal region, with hematoma volumes of 41.0 (35.0, 47.5) ml, midline displacement degrees of 0.55 (0.3, 0.7) cm, and hematoma thicknesses of 6.00 (5.0, 8.0) cm. Among the 97 cases, craniofractures were present in 95 patients, subdural hematomas in 19 cases, subarachnoid hemorrhages in 20 cases, and cerebral contusions in 47 cases. Preoperative imaging data did not reveal any significant differences between the experimental and control groups.

**Table 2:** Preoperative imaging data.

Targets	Experimental group (*n* = 37)	Control group (*n* = 97)	*p*	*z/χ*^2^*/*Fisher
Hematoma site, *n*(%)				
Frontal	9(24.32)	32(32.99)	0.330	0.947
Temporal	12(32.43)	34(35.05)	0.775	0.081
Frontoparietal	9(24.32)	17(17.53)	0.347	0.792
Temporoparietal	3(8.11)	9(9.28)	0.832	0.045
Occipitoparietal	4(10.81)	6(6.19)	0.362	0.830
The amount of hematoma at admission (ml), Median (IQR)	40.0(36.5,46.0)	41.0(35.0,47.5)	0.714	−0.366
Degree of midline shift (cm)	0.44(0.3,0.6)	0.55(0.3,0.7)	0.177	−1.349
Thickness of hematoma (cm)	6.00(4.8,7.8)	6.00(5.0,8.0)	0.502	−0.672
Complication, *n*(%)				
Skull fracture	27(72.97)	70(72.16)	0.925	0.009
Subdural hematoma	12(32.43)	19(19.59)	0.115	2.485
Subarachnoid hemorrhage	10(27.78)	20(20.62)	0.380	0.770
Cerebral contusion	25(67.57)	54(55.67)	0.211	1.567

Acute epidural hematomas primarily affect the frontal and temporal regions. Preoperative imaging data did not reveal any discernible variances between the experimental and control groups.

### 3.3 Analysis of intraoperative and postoperative data

We conducted a comprehensive analysis and comparison of intraoperative and postoperative parameters between the two groups. Our findings revealed that the operation time [85.0 (72.0, 92.5) min], intraoperative bleeding volume [30.0 (20.0, 30.0) ml], postoperative rebleeding rate (0.00%), blood transfusion rate (16.22%), consumption of plasma [400.0 (300.0,720.0) ml] and blood cells [2.0 (1.5, 3.3) U], Recurrence/death rate within 30 days after surgery (0%), and duration of hospitalization [13.0 (11.0, 15.0) day] were lower in the experimental group compared to the control group. Conversely, the experimental group exhibited higher postoperative hematoma residual volume and drainage time compared to the control group. These differences were found to be statistically significant. However, there were no statistical differences observed in discharge GCS score, discharge MRS and 30-day postoperative GOS score (Table 3). Additionally, both groups showed significant improvements in preoperative clinical symptoms, including headache, dizziness, vomiting, and impaired consciousness, upon discharge.

**Table 3:** Comparison of surgical effect.

Targets	Experimental group (*n* = 37)	Control group (*n* = 97)	*p*	*z/χ*^2^*/*Fisher
Operation time (min), Median (IQR)	85.0(72.0,92.5)	155.0(126.5,178.5)	<0.001	−8.760
Intraoperative volume of bleeding (ml), Median (IQR)	30.0(20.0,30.0)	110.0(78.5,147.0)	<0.001	−8.936
Postoperative drainage time (h), Median (IQR)	87.0(76.5,98.0)	39.0(31.0,48.0)	<0.001	−8.839
Plasma (ml), Median (IQR)	400.0(300.0,720.0)	860.0(400.0,1680.0)	0.010	−2.565
Blood cell (U), Median (IQR)	2.0(1.5,3.3)	4.0(3.0,6.3)	0.019	−2.337
Hospitalization time, (day) Median (IQR)	13.0(11.0,15.0)	17.0(15.0,21.5)	<0.001	−5.591
Discharge GCS, Median (IQR)	14.0(13.0,15.0)	14.0(13.0,15.0)	0.308	−1.020
Discharge MRS, Median (IQR)	2(1,2)	2(1,2)	0.269	−1.106
30-day postoperative GOS, Median (IQR)	4.0(4.0,5.0)	4.0(4.0,5.0)	0.253	−1.144
Postoperative hematoma residual volume (ml) Median (IQR)	19.0(14.5,22.5)	8.0(6.0,11.0)	<0.001	−7.670
Postoperative degree of midline shift (cm), Median (IQR)	0.18(0.13,0.31)	0.23(0.14,0.33)	0.527	−0.633
Postoperative thickness of hematoma (cm), Median (IQR)	4.00(3.00,5.75)	4.50(3.00,5.75)	0.564	−0.578
Complication, *n*(%)				
Surgical incision infection	3(8.11)	7(7.22)	0.861	0.031
Epilepsia	3(8.11)	11(11.34)	0.584	0.299
Encephaledema	4(10.81)	10(10.31)	0.932	0.007
Pulmonitis	4(10.81)	21(21.65)	0.150	2.073
Deep vein thrombosis in the lower limbs	4(10.81)	11(11.34)	0.931	0.008
Blood transfusion, *n*(%)			0.015	5.910
Yes	6(16.22)	37(38.14)		
No	31(83.78)	60(61.86)		
Rebleeding rate, *n*(%)			0.015	5.963
Yes	0(0.00)	14(14.43)		
No	37(100.00)	83(85.57)		
Reoperation, *n*(%)			0.187	Fisher
Yes	0(0%)	6(6.2%)		
No	37(100%)	91(93.8%)		
Recurrence/death within 30 days after surgery			0.011	Fisher
Yes	0(0%)	14(14.4%)		
No	37(100%)	83(85.6%)		

The experimental group exhibited lower operation time, intraoperative bleeding volume, postoperative rebleeding rate, blood transfusion rate, plasma and blood cell usage, recurrence/death within 30 days after surgery, and hospitalization duration compared to the control group. Additionally, the experimental group showed higher postoperative hematoma residual volume and postoperative drainage time compared to the control group. These differences were statistically significant, while there was no statistical variance in discharge GCS score, discharge MRS and 30-day postoperative GOS score.

## 4 Discussion

A blood clot forming between the dura mater and the skull was termed an AEDH. Blood accumulation could lead to increased intracranial pressure, ischemia, and hypoxia of brain tissue, potentially causing damage and impairing brain function, thus risking the patient’s life. The primary treatment for individuals with AEDH was emergency craniotomy, the standard procedure aimed at preventing mortality (13, 14). Craniotomy offered surgeons a broad field of view and ample operating space, enhancing the chances of alleviating the hematoma’s compressive effect and enabling complete hematoma removal, thus improving patient prognosis. However, this approach was traumatic, involving prolonged surgery and significant intraoperative bleeding, which posed clear risks to the patient. Challenges such as brain edema and obscured surgical fields complicated achieving complete intraoperative hemostasis, increasing the likelihood of postoperative rebleeding. Consequently, patients eligible for craniotomy but presenting advanced age, serious underlying illnesses, or intolerance to general anesthesia were not considered suitable candidates for this more invasive procedure. Reports also suggested potential contralateral delayed hematoma or AEDH recurrence after craniotomies for AEDH treatment. In cases of bilateral epidural hematomas or systemic injuries, the risk associated with craniotomy was high, with an unfavorable prognosis (11, 15, 16). Furthermore, patients left with a large incision post-craniotomy might experience adverse psychological symptoms such as anxiety and depression, potentially affecting their social and familial lives negatively. Consequently, neurosurgeons felt compelled to explore more effective and safer surgical techniques for managing this condition.

Drilling and drainage have recently emerged as a therapeutic option for cranial-cerebral issues, as an increasing number of neurosurgeons focus on minimally invasive approaches to treating craniocerebral injuries. The minimally invasive technique of cranial drilling and drainage, either alone or with urokinase injection, has shown favorable clinical outcomes. Liu et al. (17) first proposed drilling drainage in 2006 for 13 AEDH patients, with 11 showing satisfactory healing post-surgery, while 2 experienced hematoma re-expansion. Interestingly, in 2008, Liu et al. (18) utilized drilling drainage along with urokinase in treating 21 AEDH patients, with 18 exhibiting good recovery after surgery, while 3 did not. Drilling drainage involves smaller incisions and cranial openings compared to procedures requiring large bone windows for hematoma removal. This results in reduced intraoperative bleeding, tissue damage, surgical trauma, and recovery time, making it a more acceptable option, particularly for patients unable to tolerate general anesthesia (19). The numerous advantages justify drilling and drainage as a suitable choice for AEDH treatment. However, several authors have highlighted the risk of rebleeding post-cranial drilling and drainage (17, 18). While drilling and drainage effectively evacuate the hematoma, they do not address the source of bleeding. Further surgical intervention becomes necessary if bleeding recurs. Identifying and successfully controlling the bleeding source pose critical challenges in the drilling and drainage treatment of AEDH.

In AEDH, hemorrhage resulting from ruptured middle meningeal artery (MMA), venous sinuses (such as the superior sagittal sinus and transverse sinus), or diploic veins, all triggered by high-energy trauma, can lead to hematoma formation in the epidural space. Eighty-five percent of these hematomas are of arterial origin. Given that the MMA is situated 2 millimeters from the outer and 8 millimeters from the inner layers of the dura mater, blood from a ruptured MMA is more likely to reach the epidural space and cause a hematoma. Consequently, a sudden deterioration in the patient’s condition often occurs due to MMA tear (20, 21). Controlling bleeding from the MMA becomes crucial for stabilizing the patient’s condition, effectively halting bleeding, and preventing rebleeding. How then can one effectively control MMA hemorrhage? As interventional therapy has rapidly advanced, endovascular intervention has gained popularity for treating various illnesses. Its advantages include minimal complications, short recovery periods, minimal surgical trauma, and precise localization. Endovascular treatments have proven effective in managing conditions such as hemangiomas, aneurysms, arteriovenous malformations, lung and liver cancers, among others. Consequently, attempts at endovascular intervention in AEDH have been made. Suzuki et al. (22) initially proposed the use of endovascular interventional embolization and angiography for AEDH treatment in 2004. Experts have shown that this technique effectively addresses the bleeding source of the epidural hematoma. In 2009, a case was reported involving a patient with recurrent epidural hematoma after multiple craniotomies. This patient did not experience further hematoma expansion or rebleeding following MMA embolization (11). This highlights MMA embolization as the preferred choice for achieving hemostasis and preventing blood reaccumulation in the epidural space.

Selecting the appropriate puncture access and achieving successful skin penetration are critical for effective MMA embolization. An innovative approach to endovascular intervention, both anatomically and physiologically, involves puncturing at the anatomical snuffbox distal to the radial artery. Several research studies have suggested that transradial artery puncture may offer even greater advantages when accessed through the anatomical snuffbox. This approach has the potential benefits of reducing hemostasis time, lowering the risk of hand ischemia, preserving the proximal radial artery for future endovascular procedures or bypass, and allowing for repeated radial artery punctures (23, 24). Additionally, the carpal bones provide a parallel surface at the distal radial artery compared to the traditional proximal radial artery approach, ensuring effective and safe compression. When performing a left radial artery puncture, accessing the anatomical snuffbox offers the convenience of bringing the left hand closer to the right groin, enhancing comfort for both the patient and the operator. For patients unable to externally rotate their wrists, selecting the anatomical snuffbox as the site for radial artery puncture opens up greater possibilities. Transradial distal artery puncture presents a promising alternative to standard transradial artery access, with few reported vascular complications based on current information (25, 26).

In an effort to remove intracranial clots while controlling all sources of epidural hemorrhage and enhancing patient prognosis, Zhang et al. (27) attempted the surgical approach of MMA embolization + cranial drilling and drainage. Hemorrhage ceased immediately after embolization in 23 patients, and at the 6-month follow-up, there was no recurrence of AEDH, with all patients showing satisfactory prognoses. In this study, previous experiences were summarized, and the spring coil was chosen as the embolization material with the nasopharyngeal fossa as the puncture point for performing MMA embolization combined with drilling and drainage to treat AEDH. After a thorough evaluation of the patient’s condition, we chose several experienced surgeons to perform the surgery, and strictly adhered to the surgical protocols to prevent possible vascular injury or rupture during the treatment process. The ophthalmic and the petrous branches of the middle meningeal artery were carefully identified during the operation, as well as the presence of dangerous anastomotic branches to avoid false embolization. The findings revealed that the embolization group required less surgery time than the craniotomy group and experienced reduced intraoperative hemorrhage. Consequently, similar to previous research, the embolization group showed lower rates of postoperative rebleeding and transfusion, consumed fewer plasma and blood cells, and had shorter hospital stays than the craniotomy group. Although there were ultimately no significant differences between the two groups in terms of common postoperative complications (e.g., surgical incision infection, epilepsy, cerebral edema, pneumonia, lower extremity venous thrombosis, etc.), GCS scores at discharge, and 30-day postoperative GOS scores, there was a notable decrease in the rebleeding rate and hospitalization time, contributing to the conservation of medical resources. In this study, MMA trunk embolization was performed in all patients without preserving the rocky branch. However, in the postoperative period, no facial nerve dysfunction was observed and no other complications related to embolization were found. The reasons for this need to be further explored. These findings adequately demonstrate that TRA embolization of MMA in conjunction with minimally invasive drilling drainage is a safe and practical therapy option for AEDH. However, this study also has limitations. Firstly, it is a retrospective study, so surgical bias cannot be excluded. Moreover, it is a small-sample single-center study with a limited sample size, which necessitates validation by large-scale clinical trials in the future. Additionally, potential limitations of anatomical snuffbox puncture in tall and short patients need to be considered. The unique learning curve and complex anatomy of the distal transradial artery approach also present challenges for clinicians (28, 29). Therefore, to mitigate ischemic injury and ensure the highest safety standards for puncture, surgical operators need to be proficient in the anatomical landmarks of the distal radial artery, its three-dimensional structure, and its surrounding anatomical structures. Ultrasound-guided puncture is feasible when necessary to improve the first puncture rate, reduce the time to access the pathway, and decrease the incidence of adverse vascular events (30). In addition, the presence of dangerous anastomotic branches should first be noted during embolization. If there is traffic between the meningeal artery and the ophthalmic artery, a high degree of vigilance should be exercised. Avoid inadvertent embolization of the ophthalmic artery, resulting in visual impairment (31).

## 5 Conclusion

In conclusion, the efficacy of TRA embolization MMA combined with minimally invasive drilling and drainage in the treatment of AEDH is higher than that of traditional craniotomy treatment. Not only can it effectively control the condition of most patients, but it is also precise, less invasive, and promotes faster recovery, thus making it a viable option for clinical treatment.

## Data Availability

The raw data supporting the conclusions of this article will be made available by the authors, without undue reservation.
